# Genomic Data Describe Population Structure but Struggle to Estimate Directed Connectivity Networks

**DOI:** 10.1111/1755-0998.70174

**Published:** 2026-07-28

**Authors:** Camille Sant, Didier Forcioli, Cécile Fauvelot, Jean‐Olivier Irisson

**Affiliations:** ^1^ Laboratoire d'Océanographie de Villefranche, LOV Sorbonne Université, CNRS Villefranche‐sur‐Mer France; ^2^ Université Côte d'Azur, CNRS, INSERM Institute for Research on Cancer and Aging, Nice (IRCAN) Nice France; ^3^ LIA ROPSE Laboratoire International Associé Université Côte d'Azur ‐ Centre Scientifique de Monaco Nice France; ^4^ Université Côte d'Azur Institut Fédératif de Recherche ‐ Ressources Marines (IFR MARRES) Nice France; ^5^ UMR ENTROPIE (IRD‐Université de La Réunion‐CNRS‐Ifremer‐Université de Nouvelle‐Calédonie) Noumea France

**Keywords:** benchmark, connectivity, genetic inference, individual‐based simulations, migration rate, molecular ecology, networks analysis, population genomics

## Abstract

The movement of individuals between fragmented populations, known as population connectivity, plays a crucial role in population persistence, genetic structure and, ultimately, the definition of a species distribution range. Advances in molecular biology have made genomic data readily accessible, even for non‐model species, and they are increasingly used to estimate connectivity networks through diverse methods, including gene flow inference, clustering of individuals, and parentage assignment. Yet, no systematic study has evaluated the reliability of these different approaches in accurately estimating connectivity networks. To address this gap, we generated 3240 simulated SNP datasets based on a simple connectivity network of three virtual populations linked by unidirectional migration of known intensity. We then applied a range of analytical tools to these datasets to infer connectivity and evaluated their ability to accurately reconstruct the underlying connectivity network. We compared the accuracy of connectivity network estimates across methods and evaluated how four key parameters (population sizes, migration rates, and the sampling efforts of both individuals and SNPs) influenced their performance. Worryingly, we found that, across all methods, reliable estimates were achievable only under a narrow and largely unrealistic set of conditions: either complete individual sampling (100%) or moderate sampling (30%) coupled with low migration rates (< 10%). With lower sampling proportions (10% of individuals), as is often the case in current practice, none of the tested methods consistently recovered the true connectivity network. Among the tested methods, BA3‐SNPs emerged as the most reliable overall, although other approaches may be preferable in cases of highly differentiated populations or very high proportions of sampled individuals. Crucially, this study highlights that increasing the number of SNPs to several thousands, as is often done in population genomic studies of non‐model organisms, does not compensate for low proportions of sampled individuals, contrary to common assumptions. These findings raise a strong cautionary flag: without substantially high individual sampling efforts, there is a high risk of drawing misleading conclusions about connectivity, potentially leading to flawed ecological and management decisions. Our results therefore call for a careful reconsideration of current standards in the design of population genomic studies on non‐model organisms aimed at inferring connectivity networks.

## 1 Introduction

Population connectivity (i.e., the exchange of individuals between geographically fragmented populations) has a major impact on many processes, such as population regeneration and persistence, genetic differentiation, and local adaptation (Fahrig and Merriam 1985; Kawecki and Ebert 2004). In a fragmented landscape, isolated populations are more likely to decline because they have less incoming genetic diversity to adapt to local changes and will not be repopulated by other populations in case of high mortality events. Thus, accurate and reliable estimates of connectivity are crucial to understand population structure, adaptation scales and enable effective biodiversity conservation programs or management of invasive species (Olds et al. 2012; Tamburello et al. 2019). A wide variety of techniques are used to assess connectivity, some relying on empirical approaches such as capture‐mark‐recapture or population genetics and others on modelling such as Lagrangian dispersion models (Kool et al. 2012). The technique chosen is determined by the application purpose of the study but also largely by the type of data available.

Among those techniques, population genetics approaches are among the most widely used and allow estimating connectivity of species that, for example, cannot be physically tagged (Hedgecock et al. 2007). They rely on the fact that migration, and the associated transfer of genetic material between subpopulations, shapes the geographic distribution of genetic diversity (Slatkin 1987). Thus, studying this distribution provides a valuable proxy to estimate connectivity. However, population genetics approaches come with their own set of challenges and limitations. Firstly, sampling is often limited to a small number of individuals within a population, which may not fully represent its genetic diversity (Bashalkhanov et al. 2009). Secondly, the genetic structure of a metapopulation is not affected by migration only: it results from the effective transfer of genetic material between subpopulations, which is also impacted by population size and by the survival and subsequent reproduction of migrants (Lowe and Allendorf 2010; Slatkin 1987). Finally, genetic connectivity can be estimated from different types of genetic markers (such as microsatellites and Single Nucleotide Polymorphisms, or SNPs) and with different methods, each of which can reveal different aspects of the metapopulation's genetic structure (Zimmerman et al. 2020; Lowe and Allendorf 2010).

In the face of accelerating biodiversity loss, the need for robust population genomic tools in wildlife biology and conservation has never been greater (Hohenlohe et al. 2021). As the field transitions from traditional genetic markers to genomic‐scale data, single nucleotide polymorphisms (SNPs) have become the markers of choice for non‐model organisms, where reference genomes are often lacking and resources are limited (Andrews et al. 2016; O'Leary et al. 2018). Reduced‐representation sequencing methods, such as RADseq and genotyping‐by‐sequencing (GBS), have made it feasible to generate SNP datasets at relatively low cost, enabling researchers to access genome‐wide variation across a wide range of taxa (Davey et al. 2011; Elshire et al. 2011). These approaches are supported by increasingly streamlined lab protocols and accessible bioinformatic pipelines (Catchen et al. 2013; Rochette and Catchen 2017). Compared to microsatellites, SNPs offer broader genomic coverage, greater reproducibility, and higher statistical power for detecting subtle population structure and demographic patterns (Allendorf et al. 2010; Helyar et al. 2011; Morin et al. 2004). Empirical studies have demonstrated that SNPs can outperform microsatellites in resolving fine‐scale structure and estimating connectivity in conservation‐relevant contexts (e.g., Zimmerman et al. 2020; Hodel et al. 2017; Hauser et al. 2021), and they can be successfully genotyped even from low‐quality or noninvasively collected samples using platforms such as microfluidic arrays (Von Thaden et al. 2017). As a result, SNPs are now widely used in conservation genetics to delineate populations, assess gene flow, and inform species management strategies (Shafer et al. 2015; Supple and Shapiro 2018). However, practical limitations—such as budget constraints, low DNA yield, and restricted sampling opportunities—often result in datasets containing 1000 to 20,000 SNPs, and 150–500 individuals, particularly for non‐model species (e.g., Wray et al. 2025; Macleod et al. 2024; Widhelm et al. 2021; Galaska et al. 2021; Meek and Larson 2019; McCartney‐Melstad et al. 2019). It is important to note that the amount of information contained in these datasets depends not only on the number of SNPs, but also on the levels of missing data and SNP variability. This raises critical questions about the reliability and resolution of connectivity estimates under such data constraints—an issue of growing importance for genomic‐informed conservation.

To infer the degree of connection between populations or sampled sites, simple approaches, such as estimating the number of migrants exchanged per generation (Nm) based on *F*_ST_ (Hedgecock et al. 2007), have historically been used. While such metrics provide a useful summary of overall genetic differentiation, they rely on equilibrium assumptions and therefore reflect long‐term, averaged migration patterns. Moreover, they do not identify which populations exchange migrants with which others, nor the directionality of these exchanges. Yet, for many ecological and conservation questions, assessing contemporary connectivity and discriminating sources and sinks is essential (Kool et al. 2012).

In this context, connectivity is more appropriately described through a “network” framework. A connectivity network is defined as a directed and weighted graph in which nodes represent metapopulation patches (or, more simply, “populations”), directed edges (A→B and B→A) represent the existence and direction of connectivity between them, and weights associated with edges represent the intensity of that connectivity (Bode et al. 2008; Shtilerman and Stone 2015; Jones and Manseau 2022). Such networks can be decomposed into two complementary components: (i) the network structure describes the presence, absence, and direction of connections between populations (i.e., a binarized but directed network, which can equivalently be represented as a binary, non‐symmetrical adjacency matrix), addressing the question “how are populations connected?”; (ii) the weights values quantify migration intensity, addressing the question “how strongly are populations connected?”. In practical terms, this means that reconstructing connectivity involves both identifying which populations are connected (i.e., the presence and direction of edges) and estimating the strength of these connections (i.e., edge weights). For instance, missing a connection (or, conversely, inferring a spurious one) corresponds to removing (or adding) an edge, whereas misestimating migration intensity corresponds to assigning incorrect weights to existing edges.

Inferring such connectivity networks from genomic data requires more advanced population genetics methods than historical equilibrium‐based approaches. Such methods rely either on the direct estimation of migration rates or on the detection of migrants and their population of origin (Manel et al. 2005). Each method provides different types of information on recent exchange among populations (e.g., migrant detection, ancestry proportions, or gene flow estimates), which can be represented within this common directed network framework. These methods can be broadly classified into three groups (Broquet and Petit 2009). Gene flow methods, as defined here, gather tools that are used to derive population‐level connectivity from genetic data, by directly producing population‐to‐population migration rates or proxies. In all cases, the resulting outputs are used as directional and weighted measures of connectivity between populations, without relying on an explicit post hoc detection of individual migrants (Wilson and Rannala 2003; Mussmann et al. 2019; Sundqvist et al. 2016). Clustering methods gather individuals based on genetic similarity (Jombart et al. 2010; Frichot et al. 2014) allowing the detection of first‐generation migrants and the post hoc derivation of recent migration rates by aggregating individual assignments at the population level (Paetkau et al. 2004; Manel et al. 2005; Broquet and Petit 2009). Parental assignment methods identify parent‐offspring pairs among sampled individuals (Huisman 2017; Manel et al. 2005), which, like clustering approaches, enables the detection of first‐generation migrants and the underlying connectivity network (Catalano et al. 2021). Although not initially developed to estimate connectivity, clustering and parentage assignment methods are now widely used to assess the proportion of migrants exchanged between populations (Broquet and Petit 2009; Kool et al. 2012; Selkoe et al. 2016; Hedgecock et al. 2007; Manel et al. 2005). By construction, these methods estimate recent connectivity based on the detection of first‐generation migrants. As such, they reflect migration events occurring within a single generation and do not integrate the cumulative effects of migrant survival and reproductive success across multiple generations. In contrast, gene flow‐based approaches estimate migration over several recent generations; even methods specifically designed to infer contemporary gene flow, such as BA3‐SNPs, integrate information across at least two generations.

Although all three groups of methods are increasingly used to infer connectivity networks (beyond simply describing the genetic structure), little attention has been given to the factors influencing their reliability. In a recent paper, Schiebelhut et al. (2024) noted that “it remains unknown how the interactions between genome‐scale samples of loci and individuals impact the ability to infer migratory rates” and emphasized the need for simulation studies to address this gap. In response, our study evaluates the ability of commonly used genetic methods to reconstruct connectivity networks from genomic data. Importantly, errors in inferred connectivity networks can arise from two distinct sources: (i) errors in network structure, when connections are incorrectly inferred (omitted, added, or misoriented), and (ii) errors in edges weights, when the strength of migration is misestimated even if the correct structure is identified. Here, we aim to explicitly account for these two types of errors, as structural errors can have disproportionate consequences for connectivity‐based inference and management decisions. To achieve this, we simulated SNP datasets across a range of demographic scenarios and sampling intensities, applied commonly used inference methods, and compared the resulting connectivity networks to the true, simulated networks using a custom performance metric that integrates both structural and intensity errors and provides insight into their relative contribution. Finally, we assessed how demographic and sampling factors influenced the accuracy of the inferred networks.

## 2 Materials and Methods

### 2.1 Data Simulations

**FIGURE 1:**
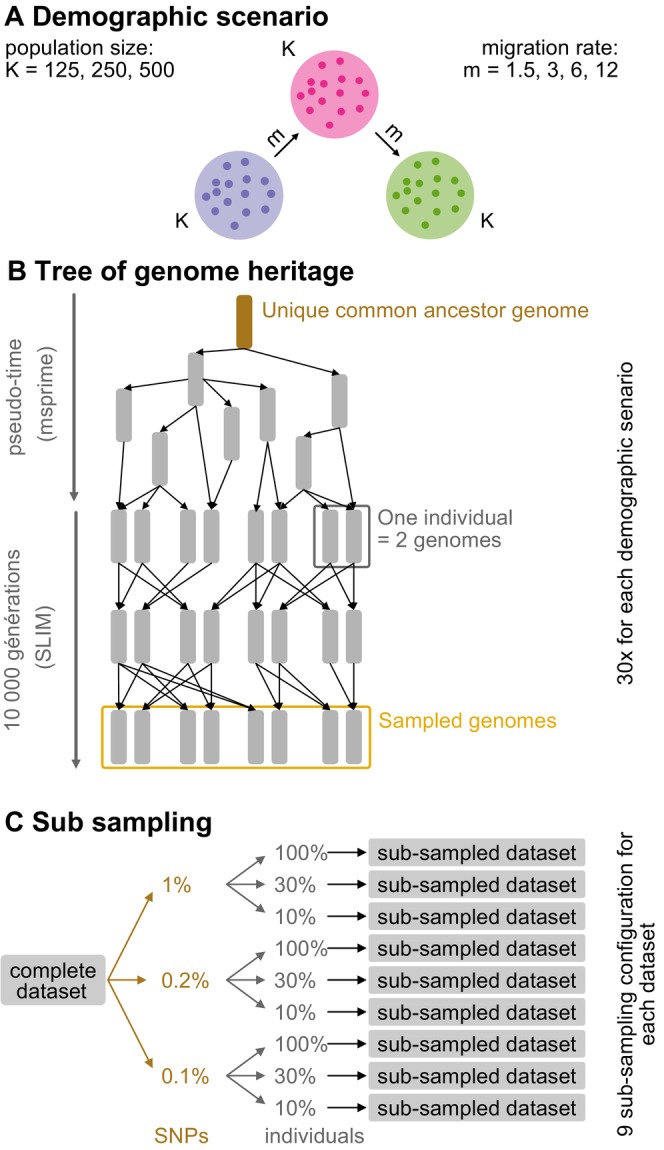
Data simulation pipeline. This chart illustrates the three steps followed in the data simulation process. (A) Demographic scenario simulation: Three populations, each with size *K*, are represented by coloured circles, and an unidirectional emigration *m* goes from the first population to the second and from the second population to the third. (B) Genome heritage simulation: Each genome copy is represented by a grey box, with an individual corresponding to two genome copies. Genome transmissions are shown by arrows. The SNPs from all the individuals at the final time step of the simulation form an unsubsampled dataset. (C) Sampling simulation: All possible combinations of sampling proportions are represented.

To evaluate the performance of the tools in a scenario where reconstructing the connectivity network should be straightforward, we simulated datasets using a simple demographic setup consisting of three populations connected by a unidirectional migration m from population 1 to population 2 and from population 2 to population 3 (Figure 1A). From this base configuration, we generated 12 different demographic scenarios by combining four emigration rates (1.5%, 3%, 6%, and 12%) with three population sizes (125, 250, and 500 individuals per population), covering a range of real‐life situations. For each scenario, trees of genome heritage were computed using SLiM v4.0.1 under a non‐Wright‐Fisher model (Haller and Messer 2019) over 10,000 generations and a msprime v1.2.0 coalescent simulation (Kelleher et al. 2016) to reach a unique common ancestor (Figure 1B). This approach allowed us to derive the genome of every simulated individual from a real reference genome (Ledoux et al. 2020), chosen to match the focal species of one of our population genetic studies, thereby providing realistic genomic data. As suggested in SLiM's documentation, a mutation rate of 10^−7^ and a recombination rate of 10^−8^ per nucleotide were applied to the genome heritage tree at each reproductive event. Each scenario was simulated 30 times to minimize potential bias due to stochastic variation.

Individuals and SNPs were subsampled from the simulated populations at the end of the simulation to reflect a range of individual sampling intensities and SNP densities (Figure 1C). For SNPs, we retained 0.1%, 0.2%, or 1% of the available loci, yielding on average 790, 1510, and 5908 SNPs, respectively, after applying the filtering steps described below. Drawing upon Nazareno et al. (2017), who showed that accurate *F*_ST_ estimates are achievable with a limited number of individuals provided that enough (> 1500) SNPs are available, our experimental design enabled us to explore whether this compensation effect also occurs for the estimation of connectivity. Specifically, retaining 0.1% of SNPs resulted in a low marker density (790 SNPs), 0.2% closely approximated the 1500‐SNP threshold (1510 SNPs), and 1% provided a high marker density (5908 SNPs), within the range commonly used to infer population structure and connectivity in non‐model organisms (e.g., Wray et al. 2025; Macleod et al. 2024; Widhelm et al. 2021; Galaska et al. 2021; Meek and Larson 2019; McCartney‐Melstad et al. 2019). Because low‐minor‐allele‐frequency SNPs are commonly discarded in real‐world datasets to reduce the risk of including sequencing errors, we only retained biallelic SNPs with minor allele frequencies above 0.02. Several downstream analyses, such as Sequoia and BA3‐SNPs (more specifically BayesAss, on which it is based), explicitly assume independence among loci (Wilson and Rannala 2003; Mussmann et al. 2019). Others, like sNMF, are known to be sensitive to linkage disequilibrium, and LD filtering is recommended to minimize potential biases (Frichot et al. 2014). To avoid such biases and ensure comparability across methods, we pruned SNPs to retain only one variant per LD group (*r* ^2^ > 0.2). For individuals, subsampling was performed at the final simulation time step across all age classes present, ensuring overlapping generations and allowing parent‐offspring pairs to occur in the sampled datasets. Sampling proportions of 10%, 30%, or 100% were applied to the full population present at that time. Because population sizes slightly fluctuate around targeted values due to density‐dependent regulation, the exact number of sampled individuals varied marginally among replicates. Consequently, the number of sampled individuals was rounded to the nearest integer when needed. Although empirical studies typically rely on fixed sample sizes, proportional sampling was used here to control the relative amount of individual‐level information across population sizes and to facilitate method comparisons. While sampling 100% of individuals is rarely feasible in real‐world studies, this scenario served as a benchmark for assessing method performance under optimum conditions. If a method failed to accurately reconstruct the simple simulated connectivity network even with complete individual sampling, it clearly indicated the method was unreliable. In total, this framework generated 3240 simulated datasets. The pipeline used to generate the final VCF files (including the orchestration of SLiM demographic simulations, the generation of genetic data using msprime, and the subsampling of individuals to mimic empirical sampling effort and of SNPs to reflect sequencing density) was implemented in Python 3.9. Subsequent SNP filtering steps, designed to mimic standard bioinformatic processing, were implemented in R 4.4.1. Additional details on the simulation parameters are available in the Table S1. As a quality control step, we verified that the simulated data followed expected population genetic patterns: genetic differentiation between populations (global *F*_ST_) decreased with increasing population size and emigration rate, while within‐population deviation from random mating (*F*_IS_) remained stable and gene diversity (H_S_) increased under the same conditions. These metrics were estimated using the hierfstat v0.5–11 R package, averaging values across loci and the three simulated populations.

### 2.2 Methods to Estimate Connectivity Networks

We selected eight methods to estimate connectivity networks, ensuring a comprehensive representation of the three main categories of approaches: gene flow, clustering, and parentage assignment. To ensure efficient application across the large number of datasets, we selected only methods that provide a command‐line interface.

As explained in the introduction, gene flow estimation methods infer connectivity patterns from genetic data by estimating directional migration rates or related proxies derived from allele frequency patterns across populations. We selected three programs in this category, based on their ability to handle large SNP datasets, their performance in past studies, and their focus on reconstructing recent gene flow.

- **BA3‐SNPs** (Wilson and Rannala 2003; Mussmann et al. 2019) uses a Bayesian framework to estimate recent migration rates, population inbreeding coefficients, individual ancestry, and population allele frequencies. All parameters are estimated simultaneously via Markov Chain Monte Carlo. To minimize user‐driven decision bias and ensure that results reflect the default behaviour of the method rather than subjective parameter choices, mixing parameters were automatically adjusted using BA3‐SNPs‐autotune and the numbers of generations were kept to their default values (a total of 1000,000 MCMC generations with a burn‐in of 100,000 generations). Migration rates estimated by BA3‐SNPs were used directly as measures of connectivity.
- **divMigrate** (diveRsity v1.9.90; Sundqvist et al. 2016) detects asymmetric migration between pairs of populations. It constructs a hypothetical migrant pool by combining the allele frequencies of the two populations, and then measures genetic divergence between this pool and each population to estimate directional migration. Among the available outputs, we retained the statistic denoted “Nm”. By default, divMigrate normalizes these values by scaling them relative to the maximum value observed across all population pairs. Here, we disabled this normalization and instead scaled the resulting values by the simulated population size to place them on a scale comparable to that of other methods. We used 10 bootstrap iterations for each run.
- **sNMF** (LEA v3.12.2; Frichot et al. 2014) infers ancestry coefficients for every individual using sparse Non‐negative Matrix Factorization (NMF) and least‐squares optimization. We obtained population‐level admixture coefficients by averaging the ancestry coefficients of all individuals within each population. These values were used as a proxy for connectivity, reflecting the degree of genetic mixing between populations, rather than as explicit estimates of migration rates. sNMF was included because it is a fast, widely used population structure method, adapted to high‐density SNP datasets. It is used here to derive a connectivity proxy at low computational cost and to provide a comparative baseline against which more computationally demanding methods specifically designed to infer recent migration or gene flow are compared. This gave an approximation of the migration rate between populations. For each dataset, 10 runs were performed assuming 3 ancestral populations, and the run with the lowest cross‐entropy was retained.

By design, BA3‐SNPs and sNMF cannot produce migration rates of exactly zero, instead yielding very small nonzero values. As a result, every estimated output network would be fully interconnected, leading to a very poor fit of the structural component and disproportionately penalizing BA3‐SNPs and sNMF. To address this, we applied a thresholding procedure to distinguish negligible values from meaningful connections. For each inferred network, edge weights were sorted in ascending order. The differences between successive values were computed, and the largest gap in this ordered distribution was identified. This gap typically separates a group of very small values (reflecting background noise) from higher values corresponding to actual connections. All edges with weight values below this identified gap were removed by setting their weight to zero.

Clustering methods classify the individuals into groups based on their genetic similarity; usually such groups are strongly related to sampling locations when a strong population structure is observed. First‐generation migrants can then be detected as individuals assigned to a different group from the location in which they were sampled. These migrants define connectivity among populations, giving access to the connectivity network. We selected three programs in this category, each employing distinct clustering algorithms and capable of handling a large number of SNPs.

- **sNMF** (LEA v3.12.2; Frichot et al. 2014) was also used here. Individuals were assigned to the population for which they showed the highest ancestry coefficient, and migrants were identified as individuals assigned to a population different from their sampling location. This second use of sNMF differs from the one presented in the gene flow section, as it relies on individual assignment to detect first‐generation migrants rather than on population‐level aggregation of ancestry coefficients.
- **DAPC** (adegenet v2.1.10; Jombart et al. 2010) employs discriminant analysis of principal components (DAPC), which transforms the data using principal component analysis (PCA) and then performs discriminant analysis to find the clusters that maximize between‐clusters variance and minimize within‐clusters variance. We performed DAPC for a fixed number of 3 clusters, and retained 100 principal components at the PCA step. As highlighted by Jombart et al. (2010), there is currently no consensus on an optimal strategy for selecting the number of principal components in DAPC. This number thus remains somewhat arbitrary. A value of 100 is suggested in the DAPC documentation, as it generally provides good discriminative power while filtering out some of the noise present in datasets with hundreds to thousands of SNPs.
- We used **COANCESTRY** (related v1.0; Wang 2011) to compute a matrix of pairwise relatedness between individuals, following (Lynch and Ritland 1999). This matrix was then interpreted as an undirected individual‐level coancestry network (distinct from the connectivity networks evaluated in this study), where nodes are individuals and edge weights correspond to their relatedness values. Next, we applied the Louvain community detection algorithm (igraph 2.0.3; Blondel et al. 2008) to identify groups of highly connected nodes within these networks. To ensure that the algorithm identified three clusters (corresponding to the three simulated populations), we progressively removed the edges of lower weight until the Louvain algorithm highlighted three clusters.

Parentage assignment methods detect pairs of parent‐offspring among sampled individuals. Similar to clustering methods, they enable the detection of first‐generation migrants, allowing the reconstruction of connectivity networks based on these migration events. For this category, we used Sequoia (sequoia v2.11.2; Huisman 2017), a likelihood‐based method that identifies the most probable relationship (parent‐offspring, second‐ or third‐degree relationships, or unrelated) between a focal individual and all potential candidates, using a heuristic hill‐climbing algorithm to explore the likelihood surface. This tool has been used in two different ways:

- **Sequoia Duo** allowed all parent‐offspring pairs to be considered, including cases when only one parent was identified.
- **Sequoia Trio** added the requirement that both parents of the offspring be identified for the relationship to be retained.

In both cases, we used the GetMaybeRel function from the sequoia package in parent‐offspring mode. Sex and age were not provided during the parentage inference step and relationships were therefore inferred solely from genotype data. We lowered the Tfilter parameter (the minimum log10‐likelihood ratio required to consider a pair as a potential parent‐offspring) to −4, which allowed for the detection of more potential parent‐offspring pairs at the cost of increased computational time. For the Duo approach, inferred parent‐offspring pairs were subsequently oriented using sample age information to translate parentage links into directional migration events. Individuals were assigned to four age classes (0–1, 2–5, 6–10, and 11–20 years) to reflect the uncertainty typically associated with age estimation in natural populations, and the individual belonging to the older age class was assigned as the parent. When both individuals belonged to the same age class, age information was insufficient to determine directionality and these pairs were excluded. For the Trio approach, directionality does not rely on age information, as the simultaneous identification of both parents unambiguously defines parental and offspring roles.

Parameters not mentioned in this section were kept at their default values and detailed in Table S2. The code used to perform these analyses is provided at https://doi.org/10.5281/zenodo.15211977 for further details. All computations were run on a Dell Precision 7920 workstation, with Intel Xeon Gold 6240 CPUs, 192GB of DDR4 2933 MHz RDIM RAM with Error Correcting Code, running Ubuntu Linux 24.04.

### 2.3 Performance Evaluation

Consequently, methods for estimating connectivity must be both efficient and accurate. To evaluate the performance of each method, we assessed its computational efficiency (measuring runtime, memory usage, and CPU load) alongside the accuracy of its connectivity estimates.

Resource usage during analysis was recorded using psrecord (v1.4), capturing maximum CPU, memory, and runtime consumption for each run.

**FIGURE 2:**
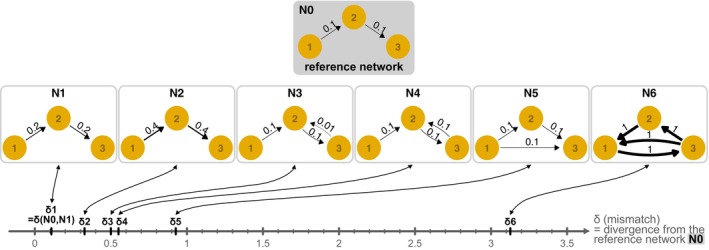
Examples of mismatch (δ) values between a reference network (N0) and a series of example networks (N1 to N6). The reference network N0 is compared with a series of example networks that increasingly deviate from N0 (from N1 to N6): Initially only edge weights change, then new connections are added, with increasing weights. The values of δ resulting from each comparison are shown on the horizontal axis.

To assess the accuracy of connectivity estimates, we represented connectivity as a directed network (with populations as nodes and edges corresponding to migration rates) and applied a network comparison approach. We quantified the dissimilarity between the connectivity network estimated by each method and the network derived from the simulations, representing the true network to be recovered by the methods (N0 in Figure 2, hereafter referred to as the reference network). Migration in the simulations is implemented stochastically and parameterized as a juvenile emigration rate, whereas the methods evaluated here infer connectivity from the genetic composition of individuals currently present in each destination population. To ensure an adequate comparison between simulated and inferred networks, the reference matrix/network was therefore constructed from recent realized immigration rates rather than from the imposed migration parameters. Specifically, at the end of each SLiM simulation, for population j comprising N_j_ live individuals, we counted the number of individuals (across all age classes) that originated from each population i and now reside in j (N_i→j_) and computed proportions N_i→j_/N_j_ for all i and j. This defines the reference matrix, which describes the population‐of‐origin composition of each destination population. This approach serves three purposes. First, it expresses connectivity in terms of immigration proportions, which corresponds directly to the quantities inferred by the evaluated methods. Second, because it is based on the pool of individuals alive at the end of the simulation, it captures recent connectivity (integrated over a few generations on the order of the life expectancy of the species, i.e., maximum 20 years in our simulations), allowing a more appropriate evaluation of the methods' ability to reconstruct recent connectivity. Third, by using realized rather than imposed rates, it reflects the actual outcome of the simulation, which may differ from theoretical expectations due to stochastic variation in simulated migration. This is particularly relevant for methods based on migrant detection or parentage assignment, which rely on first generation migrant detection and remains relevant for gene‐flow methods, as it is computed from all individuals alive at the end of the simulation and therefore integrates connectivity over several generations. We then compared these reference networks to the networks estimated by each method using a dissimilarity metric designed to satisfy the following two properties:

- P1: Larger differences in edge weight (i.e., migration intensity) lead to higher dissimilarity metric values.
- P2: Qualitative changes in connectivity (e.g., adding or removing edges) are more heavily penalized than changes in migration intensity.

To achieve this, we developed a custom metric, hereafter called the mismatch and noted δ, computed as the unweighted arithmetic mean of two components:

- **Graph Diffusion Distance (GDD)** (Hammond et al. 2013) computed on binary networks: This metric quantifies structural (qualitative) differences between networks by measuring the discrepancies in particle diffusion paths. Diffusion starts at a given node and proceeds through the binarised network (where edges are either present or absent) over a diffusion time 𝜏. The diffusion process is represented using the Graph Laplacian exponential kernel matrix, where each column corresponds to one different starting node. The structural differences between networks are then quantified by computing the Frobenius norm of the difference between the matrix of the reference network and that of the estimated network. This process is repeated over a wide range of 𝜏 values and the maximum difference is retained as the GDD. Calculations were performed using the nd.gdd function from the NetworkDistance v0.3.4 R package.
- **Scaled Mean Absolute Error (MAE)**, computed on weighted networks: This metric captures differences in migration intensity between networks in a quantitative manner. It is computed as the average of the absolute differences between the reference and the estimated networks, across all edges, divided by the mean migration rate.

Our custom mismatch metric was then computed as δ = (*GDD + MAE*)/2.

We provide examples of δ values across their distribution range to clarify the interpretation of different scores (Figure 2). Values of δ below 0.5 indicate a good representation of the true network structure, where either the correct structure is identified (N1) or an additional edge is added with a significantly lower weight than those in the reference network (N3). However, the weights of correctly identified edges can still deviate considerably from their true values (N2). Values of δ between 0.5 and 1 indicate substantial structural changes, such as the addition or removal of edges with weights comparable to those in the reference network (e.g., N4). Although δ technically ranges from 0 to infinity, for the tested configuration of three nodes and edge weights between 0 and 1, δ rarely exceeded 3 (N6).

All analyses and data visualization were performed using R v4.4.1.

## 3 Results

### 3.1 Data Quality Check

To verify that the simulated data aligned with expected trends of population genetics, we examined the variations in *F*_ST_, *F*_IS_, and gene diversity (H_S_) according to the simulated population sizes and migration rates across the 3240 subsampled simulated datasets (Figure S1). As anticipated, the mean *F*_ST_ decreased with increasing migration rates and population sizes, indicating greater genetic similarity between populations as gene flow and effective population size increased. Observed *F*_ST_ values were consistently of the same order of magnitude but slightly higher than theoretical expectations under a Wright‐Fisher island model (*F*_ST_ = 1/(4 Nm + 1); shown for reference in Figure S1), which is expected given the non‐Wright‐Fisher demography, age structure, and unidirectional step‐stone migration implemented in the simulations. In contrast, the mean *F*_IS_ remained relatively stable across migration rates and population sizes, as expected under random mating within populations, confirming that no systematic deviation from Hardy–Weinberg equilibrium was introduced by the simulation framework. Gene diversity (H_S_) increased with population size and migration rate and tended to plateau at the largest population size (*N* = 500). This reflects reduced genetic drift and increased gene flow promoting allele mixing, with the combined effects of these processes reaching a saturation point at higher values. In addition, and as also expected under standard population genetic theory, the variance in both *F*_ST_ and *F*_IS_ decreased with increasing population size, reflecting reduced stochastic variation in larger populations and further supporting the consistency of the simulations.

Globally, realized migration rates were quite variable and slightly higher than the imposed emigration rates on average: 1.75% ± 0.89% (mean ± standard deviation) instead of 1.5%, 3.26% ± 1.34% instead of 3%, 6.83% ± 1.70% instead of 6%, and 13.16% ± 2.32% instead of 12%. This difference reflects the fact that realized migration rates were computed as immigration‐based proportions in order to ensure consistency with the quantities inferred by the evaluated methods. The relatively large standard deviation allows for the exploration of a wide range of migration rates.

### 3.2 Resource Usage

**FIGURE 3:**
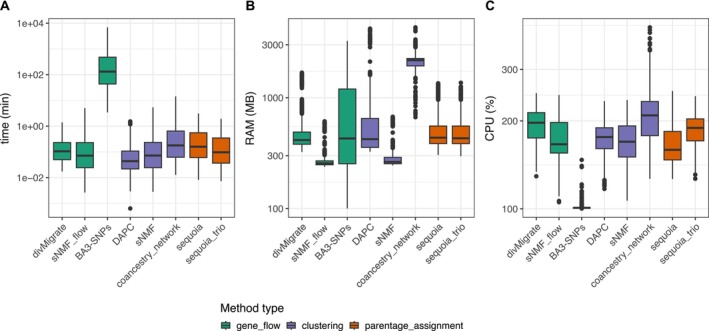
Resource usage of the eight tested methods across different metrics. Boxplots show the distribution of runtime (A), memory (B), and CPU usage (C) across simulation runs for each method with *y*‐axes displayed on a logarithmic scale. For CPU, 200% means that 100% of two cores are used. Colours indicate the broad method category: Green for gene flow, purple for clustering, and orange for parentage assignment.

The eight methods evaluated showed a wide range of resources usage in terms of computation runtime (from less than a second to several days), CPU usage (between one and four CPU cores completely used), and memory (RAM, from indetectable to more than 3Gb; Figure 3). Resource usage generally increased with both the number of SNPs and the number of individuals analysed (Figure S2). Apart from BA3‐SNPs that ran up to 5 days, all runs were completed in less than 15 min, even with the largest datasets. Overall, memory and CPU requirements were low and all methods can easily run on a standard laptop. Ranges of CPU load were similar across methods, except for BA3‐SNPs which showed significantly lower values. However, BA3‐SNPs runtime was considerably longer than that of other methods, indicating that its overall computational cost is not necessarily lower but distributed over a longer execution time. This can be explained by the fact that this method cannot be parallelized within a single run, which is an opportunity for potential improvement.

### 3.3 Overall Mismatch (δ) Distributions for Each Methods

**FIGURE 4:**
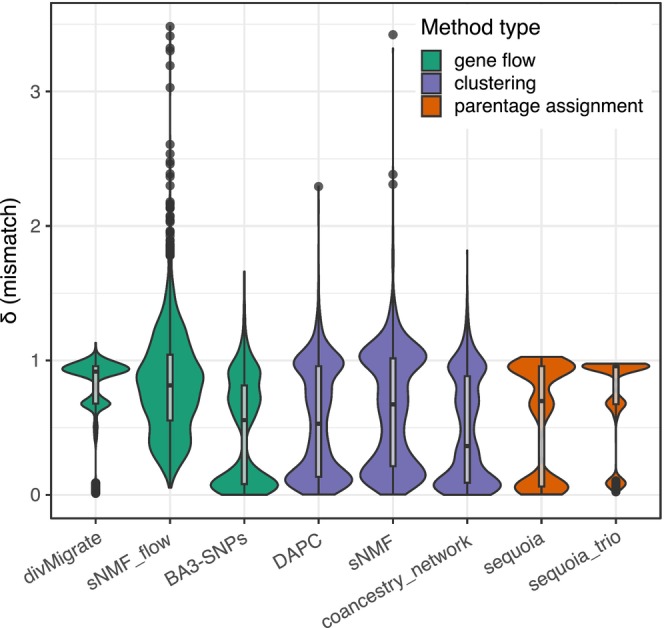
Distribution of mismatch (δ) for the eight methods. Violin and box‐plots of the distribution of δ across all demographic and sampling scenarios for each method. Colours indicate the broad method category: Green for gene flow, purple for clustering, and orange for parentage assignment.

Across all demographic scenarios and sampling proportion (of individuals and SNPs), δ between the estimated and true connectivity networks ranged from 0 to 3.48 (Figure 4). Mean and median δ per method ranged from 0.47 to 0.84 and 0.36 to 0.96 respectively, with 45% to 96% of δ values exceeding 0.5. As illustrated by the examples shown in Figure 2, a δ around 0.3–0.5 corresponds to networks with correct structure but substantial misestimation of migration intensity. Higher δ (> 0.5) are associated with increasingly strong alterations in network structure. These results indicate that, overall, the methods often failed to accurately capture the true connectivity networks.

Despite these overall high δ values, differences among methods were apparent. BA3‐SNPs, clustering methods, and Sequoia in default duo mode showed a high density of low δ, whereas divMigrate, sNMF in flow mode, and Sequoia in trio mode tended to produce mainly high δ. The coancestry network method exhibited both the lowest mean δ and the lowest proportion of δ exceeding 0.5, whereas divMigrate showed the highest values for both statistics. However, substantial overlap among distributions indicates that no method consistently outperformed all others across all scenarios.

Furthermore, for all methods, δ followed a broad, multimodal distribution, with standard deviations ranging from 0.18 to 0.42. Variance decomposition showed that differences among demographic and sampling scenarios explained between 9% and 55% of this variance, depending on the method. This reflects the fact that method performance varies strongly both across replicates within the same scenario and across scenarios, with some conditions leading to accurate network reconstruction and others resulting in substantial errors. This motivated the conditional analyses presented below.

### 3.4 Influence of Simulation Parameters on the Mismatch (δ)

To examine how simulation parameters affected the mismatch between estimated and true connectivity networks, we performed an ANOVA with δ as the response variable. This analysis was used in a comparative framework to summarize the relative importance of demographic and sampling parameters and identify the main drivers of δ, rather than to model detailed functional relationships. The explanatory variables included the method used, demographic parameters (migration rate and population size), sampling proportion (of sampled individuals and SNPs), and all their two‐way interactions. Outliers values of δ were identified using the IQR criterion and excluded for this analysis (29 points were removed among 25,920). Residual diagnostics indicated no major deviation from normality (Figure S3).

The total variance explained was 34.49%. Within this, 12.06% was explained by the method used, 7.78% by the proportion of sampled individuals, 5.6% by the interaction between the method and the proportion of sampled individuals, 1.3% by the migration rate, and 3.35% by the interaction between the method and the migration rate. The remaining parameters together explained 2.39% of the total variance, with each remaining parameter (including population size, SNP sampling proportion, and all unlisted interactions) contributing less than 1% (Figure S4). Although population genetic structure is classically expected to depend on Nm, our focus here is on estimating connectivity and, in that case, migration rate (m) explains a much larger proportion of the variance of δ than population size (N) or its interaction with migration rate (N and m combined). This indicates that the primary factors influencing δ are the method used, the proportion of sampled individuals, and the migration rate. Accordingly, the remainder of this study will focus on how these three parameters affect δ.

### 3.5 Mismatch (δ) Variations According to Sampling Proportion of Individuals and Migration Rate

**FIGURE 5:**
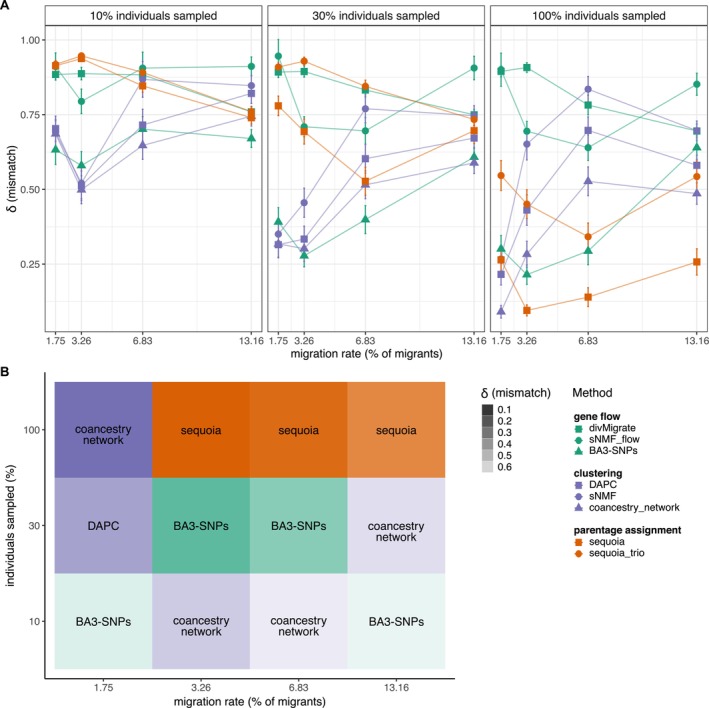
Mismatch (δ) of eight methods across individuals sampling proportions and migration rates. Colours indicate the broad method category: Green for gene flow, purple for clustering, and orange for parentage assignment. (A) δ (*y*‐axis) is shown as a function of migration rate (*x*‐axis) and percentage of sampled individuals (panels from left to right). For each combination of method, migration rate, and sampling proportion, the mean δ across the 270 simulations is represented by a point and the 95% confidence interval by a bar. The shape of the dots represents the method. (B) For each combination of migration and sampling proportions, the method with the lowest mean δ is identified. Colour intensity is inversely proportional to δ: Darker colours indicate more reliable results.

Overall, δ decreased strongly as the percentage of sampled individuals increased (Figure 5A, panels from left to right). When 10% of the individuals were sampled, average δ per method ranged from 0.50 to 0.95. As more individuals were sampled, δ globally decreased although the magnitude of this improvement varied among methods: mean δ per method ranged between 0.28 and 0.95 at 30% and between 0.09 and 0.91 at 100%. This indicates that increasing the proportion of sampled individuals improved the reliability of estimates for some, but not all, methods. The variance among simulations, quantified by the confidence interval, was much lower than the change between simulation parameters.

Conversely, δ tended to increase with migration rates for most methods (Figure 5A, from left to right within each panel). Clustering methods, in particular, showed a strong increase in δ as migration rates rose, except when only 10% of individuals were sampled. In this case, very low migration rates resulted in poorer performance than intermediate ones, as the methods failed to detect any migrants. For most other methods, δ was lowest at migration rates of approximately 3% to 6%, regardless of sampling proportions, but began to increase beyond this threshold.

Individually looking at each component (GDD or MAE) on which δ is based (Figure S5), we observed that the trends followed by δ were very similar to those of their structural component (GDD) and seemed to match less closely their migration intensity component (MAE). The MAE computed on weighted networks rarely exceeded 1. Because this metric is normalized by the mean simulated migration rate, values below 1 indicate that the error in the estimation of the migration rate is lower than its average value. In other words, the estimated migration rates were often of the correct order of magnitude. In contrast, GDD computed on binarized networks often showed values above 1 (and greater variability across sampling and migration rates), revealing that the methods often struggled to correctly identify the network structure. Investigating the effect of the proportion of sampled individuals, we found that when 10% of the individuals were sampled, only 19% of the estimated networks had the correct structure. As the sampling proportion increased, accuracy improved: with 30% of individuals sampled, the accuracy reached 33%, and with 100% of individuals, it reached 49%. This shows that while migration intensity estimates were generally close to the true values, correctly identifying the network structure remained challenging. However, increasing the proportion of sampled individuals improved the ability of the methods in this respect. Overall, the final values of δ were primarily influenced by the structural component, with the intensity component modulating the final results.

We identified the most accurate method for each combination of individuals sampling proportion and migration rates (Figure 5B). When all individuals were sampled and the migration rate was high enough to guarantee sampling of parent‐offspring pairs indicative of migration events, Sequoia in duo mode was found to be the most accurate method. In all other scenarios, the most effective methods were either BA3‐SNPs or the coancestry network. Notably, when 30% of individuals were sampled and the migration rate was ~1.75%, DAPC was the most accurate method, though its performance was nearly identical to that of the coancestry network. Overall, the three clustering methods and BA3‐SNPs yielded comparable results, with similar values of δ and frequently overlapping confidence intervals (Figure 5A), making it challenging to identify the most accurate method.

## 4 Discussion

In this study, we evaluated a wide range of methods for inferring connectivity from high‐throughput sequencing data on simulated SNP datasets generated under diverse demographic scenarios and sampling proportions for both individuals and SNPs. We quantified the accuracy of those methods through a dissimilarity metric, calculated as the average of two metrics: the Graph Diffusion Distance (GDD) on binary networks focusing on network structure and the scaled Mean Absolute Error (MAE) depicting differences in migration intensity.

Worryingly, we found that none of the methods tested can reliably estimate both the direction and intensity of connectivity under usual, or even relatively favourable empirical conditions (e.g., sampling of 10% of the adults, corresponding to approximately 10–50 individuals per population depending on population size, and of thousands of SNPs). More specifically, the analyses showed that among all factors tested, the percentage of individuals sampled had the strongest impact on the accuracy of connectivity estimates: for all methods, the discrepancy between the estimated network and the simulated one strongly increased as the percentage of individuals sampled decreased to commonly used values (10%). Migration rates also influenced the accuracy of connectivity estimates, but to a lesser extent and differently depending on the method. Before discussing the implications of our results for method selection under the various scenarios tested, we first revisit the framework of this study and address its limitations.

### 4.1 Limitations of Our Study

This study involved several methodological simplifications aimed at isolating the effects of key parameters and ensuring a high number of replicates. While these choices were justified by our objectives, their implications deserve careful consideration.

We simulated a simplified system with three populations (125–500 individuals) connected by constant, unidirectional migration. While it does not reflect the complexity of natural systems (Galaska et al. 2021; Bernal‐Durán et al. 2024), this design was intentional: if methods struggle to recover accurate networks under such simple conditions, their performance is likely to degrade further in more realistic scenarios. Our focus was on detecting non‐symmetrical migration, a challenge for many genetic structure methods (Broquet and Petit 2009; Whitlock and Mccauley 1999). The unidirectional stepping‐stone model provided a tractable framework for testing this capacity while enabling a broad exploration of methods through extensive simulations, thereby enhancing the robustness of our findings.

Sampling effort strongly influenced method accuracy, particularly for clustering and parentage‐based approaches. We tested sampling proportions of 10% and 30%, which, for the population sizes simulated in our study, correspond to 12–150 individuals per population. These values reflect common practice and feasibility in conservation contexts: 10% (12 to 50 individuals per population) aligns with many published datasets (Widhelm et al. 2021; Galaska et al. 2021) and recent recommendations for population genomic studies (Hale et al. 2012; Aguirre‐Liguori et al. 2020) and 30% (37 to 150 individuals per population) may be attainable in well‐studied taxa. We also simulated complete sampling of individuals (100%) as a benchmark even if rarely achievable (Berry et al. 2004; Steele et al. 2019). Importantly, the correspondence between sampling proportion and absolute sample size depends on population size. In systems with much larger populations than those simulated here (e.g., some exploited fish stocks; Clarke et al. 2024; Steele et al. 2019), even small sampling proportions may still yield large absolute sample sizes, potentially mitigating the loss of accuracy observed with low sampling proportions.

The choice of performance evaluation metric also affects how connectivity estimates are interpreted (Hartle et al. 2020). We evaluated multiple metrics and ultimately selected a combined measure of network structure (GDD, computed on binarized networks) and migration intensity (MAE, computed on weighted networks), weighted equally. This combination was justified by their comparable scales and interpretability. The MAE captures differences in migration intensity well, but treats all added or removed edges equivalently, as any change in connectivity is considered a difference in edge weight. In contrast, the GDD penalizes structural and directionality discrepancies based on comparison of diffusion processes across networks, thereby capturing differences in network structure in a way that is not reflected by migration intensity alone. The combined metric ultimately emphasized the correct prediction of structure over that of precise migration rates. This emphasis is appropriate, as correctly identifying the network topology and directionality is often the primary goal in connectivity studies, while migration intensity is estimable via complementary analyses (Wright 1931; Meirmans and Hedrick 2011).

Another important consideration is the number of SNPs used to infer connectivity and their variability. Our study incorporated a range of SNP numbers commonly encountered in conservation genomics—spanning from approximately 790 to 5900 loci (McCartney‐Melstad et al. 2019; Macleod et al. 2024; Widhelm et al. 2021). Whole‐genome resequencing or capture‐based approaches can provide higher‐density genomic datasets which, if sufficiently variable, may improve connectivity inference, particularly for methods relying on allele frequency differences or subtle population structure. The mismatch (*δ*) values reported here therefore reflect method performance under moderate SNP densities and typical levels of genetic variability, and may be lower when higher‐density and more informative genomic datasets are available. However, using moderate SNP numbers allowed us to evaluate eight different inference methods across 3240 simulated datasets, a scale of analysis that would have been computationally prohibitive with larger SNP datasets due to increased runtime, memory usage, and CPU demands. This trade‐off enabled us to explore broad patterns of method performance and parameter sensitivity while maintaining computational feasibility. It also reflects typical conditions encountered in population genomics of non‐model organisms, where sequencing costs, DNA quality, or library complexity often limit marker density.

Finally, comparisons among methods are complicated by differing temporal resolutions. Assignment‐based approaches (e.g., clustering and parentage) detect recent migrants (Broquet and Petit 2009; Kool et al. 2012; Selkoe et al. 2016; Hedgecock et al. 2007; Saenz‐Agudelo et al. 2012), while gene‐flow based methods capture migration over several generations. To ensure comparability, we selected gene flow methods designed for contemporary inference (e.g., BA3‐SNPs and divMigrate; Wilson and Rannala 2003; Sundqvist et al. 2016). Despite not being designed for detecting recent migration, sNMF (Frichot et al. 2014) was also included as a computationally efficient baseline, allowing us to evaluate whether more computationally demanding methods specifically developed for gene flow inference (BA3‐SNPs and divMigrate) provide substantial gains in reconstructing directed connectivity. It must however be acknowledged that sNMF integrates ancestry over multiple generations, probably overestimating recent migration rates. Since migration was parameterized with constant direction and intensity, realized migration rates (although stochastic) did not strongly shift between ancestral and recent generations. As a result, differences between ancestral and recent migration were limited, reducing the impact of discrepancies in temporal scope among methods.

### 4.2 Influence of the Methods on Inferred Connectivity Networks

Parentage assignment methods were found to be the most sensitive to the proportion of sampled individuals. Previous studies have shown that parentage inference can be highly accurate even with limited sampling (Harrison et al. 2013). However, our results indicate that, although individual parentage assignments are accurate, connectivity networks reconstructed from these assignments become unreliable at low sampling effort, because sparse sampling greatly reduces the probability of capturing parent–offspring pairs that reflect migration events. In contrast, when sampling is exhaustive, parentage assignment methods are highly accurate and outperform other approaches, even under high migration rates and consequently low genetic differentiation among populations. This pattern aligns with previous findings highlighting the strong potential of parentage analysis when near‐complete genotyping is achievable, as well as its robustness to low levels of population differentiation (Steele et al. 2019; Christie et al. 2017). The increased accuracy observed under full sampling can be attributed to the higher probability of identifying recent parent‐offspring relationships linking populations. However, such exhaustive sampling is rarely achievable, except in small, isolated populations (Berry et al. 2004) or managed hatchery populations (Steele et al. 2019). One previously proposed workaround to reduce sampling effort has been to rely on parent‐offspring pairs rather than full trios (Steele et al. 2019). Our results showed that while this strategy improved the accuracy of connectivity estimates under a high proportion of sampled individuals, it offered little to no advantage when sampling was limited. As such, it does not effectively reduce the overall sampling effort required. Consequently, parentage assignment methods may have limited applicability under typical field conditions, where obtaining large, well‐distributed sample sizes is often impractical. Nevertheless, they remain particularly valuable in high gene flow systems, with weak or absent genetic structure (Saenz‐Agudelo et al. 2012), in cases where populations form a continuum rather than a structured network, or even for investigating dispersal within a single population (which falls outside the scope of this study). In such cases, parentage assignment methods can provide reliable estimates of dispersal kernels (i.e., distributions of dispersal distances), even under low sampling effort (Bode et al. 2018).

Clustering methods required less intensive sampling but were found to be highly sensitive to migration rates. At low migration rates, where genetic differentiation was more pronounced, these methods consistently yielded the lowest δ, provided that some migrants were present in the sample. However, under high migration rates, when genetic differentiation declined, clustering methods failed to generate reliable connectivity estimates. This is consistent with previous studies using microsatellite data, both simulated (Cornuet et al. 1999) and empirical (Berry et al. 2004; Saenz‐Agudelo et al. 2012), which showed that assignment accuracy is high when genetic differentiation (*F*_ST_) is strong, but declines as differentiation decreases. Our findings confirm that this pattern persists with SNP data, despite their higher resolution and greater power to detect subtle genetic structure (Morin et al. 2004; Zimmerman et al. 2020). Moreover, we showed that reduced differentiation under high migration rates led to lower assignment accuracy, which in turn compromised the reliability of connectivity estimates derived from clustering methods. This does not challenge the value of clustering methods for their primary purpose (i.e., inferring population structure) but rather underscores their limitations when repurposed to estimate connectivity networks via assignment. While these methods remain effective in delineating broad genetic patterns, our results highlight that their application to infer directional gene flow or recent migration should be approached with caution, especially in systems characterized by weak genetic structure.

Gene flow methods demonstrated the greatest versatility across the range of sampling scenarios tested here. This is probably because, unlike clustering or parentage assignment approaches, gene flow methods do not depend on the presence of first‐generation migrants among the sampled individuals (Broquet and Petit 2009; Paetkau et al. 2004). Instead, they infer migration rates by integrating genetic information over several recent generations (Wilson and Rannala 2003; Sundqvist et al. 2016), thereby exploiting signals from later‐generation hybrid individuals, which are much more frequent and therefore more likely to be sampled than first‐generation migrants. This makes gene flow methods more robust under incomplete sampling. However, this strength comes with a trade‐off: gene flow methods are less sensitive to detecting very recent changes in connectivity (Samarasin et al. 2017). In natural systems, where migration rates may fluctuate across years or generations, these methods may reflect longer‐term averages, potentially obscuring short‐term demographic shifts or recent management impacts (Samarasin et al. 2017).

Interestingly, despite being explicitly designed to estimate asymmetric connectivity, divMigrate did not consistently provide more accurate connectivity reconstructions than the proxy derived from average admixture proportions inferred by sNMF. In contrast, BA3‐SNPs yielded substantially more accurate estimates of connectivity. These results illustrate a clear trade‐off between computational cost and estimation accuracy. While fast approaches can efficiently recover population genetic structure at very low computational cost, more specialized and computationally intensive methods such as BA3‐SNPs are justified when accurate estimates of oriented connectivity networks are required.

In line with previous work on BayesAss (Faubet et al. 2007), we found that migration rate influenced the accuracy of BA3‐SNPs. However, while Faubet et al. (2007) reported a decline in performance with increasing migration, our results revealed peak accuracy at intermediate migration rates (3%), with lower accuracy observed at both very low and high rates. This discrepancy likely reflects differences in methodological criteria: Faubet et al. (2007) assessed accuracy based on root mean square error (RMSE) of migration rate estimates (focusing on the intensity of gene flow), whereas our study also considered the accuracy of network structure, specifically the presence or absence of connections. At very low migration rates, the genetic signal becomes faint and difficult to detect, complicating the reconstruction of the network's structure. Despite these challenges, BA3‐SNPs consistently outperformed or closely matched the top‐performing method across scenarios, making it the most robust and reliable overall.

### 4.3 Influence of Individual Sampling on Connectivity Network Accuracy

Another key finding of this study is that, under the setup of our study, increasing the number of SNPs did not compensate for a low proportion of individuals sampled when estimating connectivity. This contrasts with previous results on population structure, where a large enough number of SNPs (≥ 1500) has been shown to offset small numbers of individuals, enabling accurate estimates of genetic differentiation even with as few as two individuals (Nazareno et al. 2017; Willing et al. 2012). In our analyses, however, accurate reconstruction of connectivity networks consistently required larger proportions of sampled individuals, while increasing SNP density (up to the maximum tested; see study limitations above) had only a limited effect on performance. These findings support recent suggestions by Schiebelhut et al. (2024), who argued that genotyping more individuals rather than more loci is likely to be more effective for estimating recent gene flow. This distinction highlights fundamental differences between estimating genetic structure and inferring individual‐level movement, with the latter being more sensitive to sampling effort.

### 4.4 Practical Guidelines for Method Selection and Conclusion

Once it is acknowledged that all methods yield inherently noisy estimates, even under relatively high sampling effort, the choice of a connectivity inference method should be guided by the targeted temporal scale, the level of population differentiation, and sampling intensity:

- When the primary objective is to reconstruct connectivity integrated over the last few generations, BA3‐SNPs emerges as the most reliable option across the tested configurations. We therefore recommend BA3‐SNPs as a primary tool for inferring connectivity, despite its higher computational time.
- When it is critical to infer connectivity at the most recent time scale possible, alternative approaches may be preferable under specific conditions:
  ○ In scenarios characterized by high population differentiation (*F*_ST_ > 0.03), clustering‐based methods, particularly coancestry networks, provide the most informative alternatives.
  ○ When genetic differentiation is lower and sampling effort is exceptionally high (ensuring to capture parent–offspring pairs), parentage assignment methods such as Sequoia are the most informative.

Overall, these results highlight the importance of matching the inference method to both the sampling design and the underlying population structure when aiming to reconstruct connectivity networks. They also suggest that connectivity estimates derived from genomic data—especially under typical constraints of sample size and marker density—should be interpreted with caution. No single method proved universally accurate across all tested conditions, raising concerns about the robustness of many previously published connectivity networks.

## Author Contributions

All authors conceived the ideas and designed methodology. C.S. performed the data simulations, analyses, prepared the figures, and the first version of the manuscript. J.‐O.I. and C.F. contributed to the interpretation of the analyses and edited the manuscript. D.F. helped with data simulations, analyses and reviewed the manuscript. All authors gave final approval for publication.

## Funding

This work was supported by Agence Nationale de la Recherche, ANR‐21‐CE02‐0017.

## Conflicts of Interest

The authors declare no conflicts of interest.

## Supporting information


**Figure S1:** Population genetic statistics across simulated datasets according to demographic parameters.
**Figure S2:** Detailed resource usage of the eight tested methods as a function of data size.
**Figure S3:** Residual diagnostics of the ANOVA model.
**Figure S4:** Relative contribution of model terms to the variance explained by the ANOVA on mismatch (δ).
**Figure S5:** Two components of the mismatch (δ) across sampling rates of individuals and migration rates.
**Table S1:** Demographic and genetic parameters used for data simulation.
**Table S2:** Parameters used for connectivity analyses.

## Data Availability

All scripts used to simulate the genomic datasets, the resulting data (excluding large master VCF files, which can be made available upon request), and all scripts used for downstream analyses are available at https://doi.org/10.5281/zenodo.15211977.
