# Supplementary material for: Genomic Data Describe Population Structure but Struggle to Estimate Directed Connectivity Networks
Source: Mol Ecol Resour. 2026 Jul 28;26(5):e70174. doi: 10.1111/1755-0998.70174 (PMC13409678; doi:10.1111/1755-0998.70174)
Supplement: Supplementary file 1 — Figure S1: Population genetic statistics across simulated datasets according to demographic parameters. Figure S2: Detailed resource usage of the eight tested methods as a function of data size. Figure S3: Residual diagnostics of the ANOVA model. Figure S4: Relative contribution of model terms to the variance explained by the ANOVA on mismatch (δ). Figure S5: Two components of the mismatch (δ) across sampling rates of individuals and migration rates. Table S1: Demographic and genetic parameters used for data simulation. Table S2: Parameters used for connectivity analyses. [file MEN-26-e70174-s001.docx]

**Genomic data describe population structure but struggle to estimate directed connectivity networks**

### **Supplementary Information**

**Contents**

**Supplementary Figures**

Figure S1: Population genetic statistics across simulated datasets according to demographic parameters.

Figure S2: Detailed resource usage of the eight tested methods as a function of data size.

Figure S3: Residual diagnostics of the ANOVA model.

Figure S4: Relative contribution of model terms to the variance explained by the ANOVA on mismatch (δ).
Figure S5: Two components of the mismatch (δ) across sampling rates of individuals and migration rates.

### **Supplementary Table**

Table S1: Demographic and genetic parameters used for data simulation.

Table S2 : Parameters used for connectivity analyses.

*
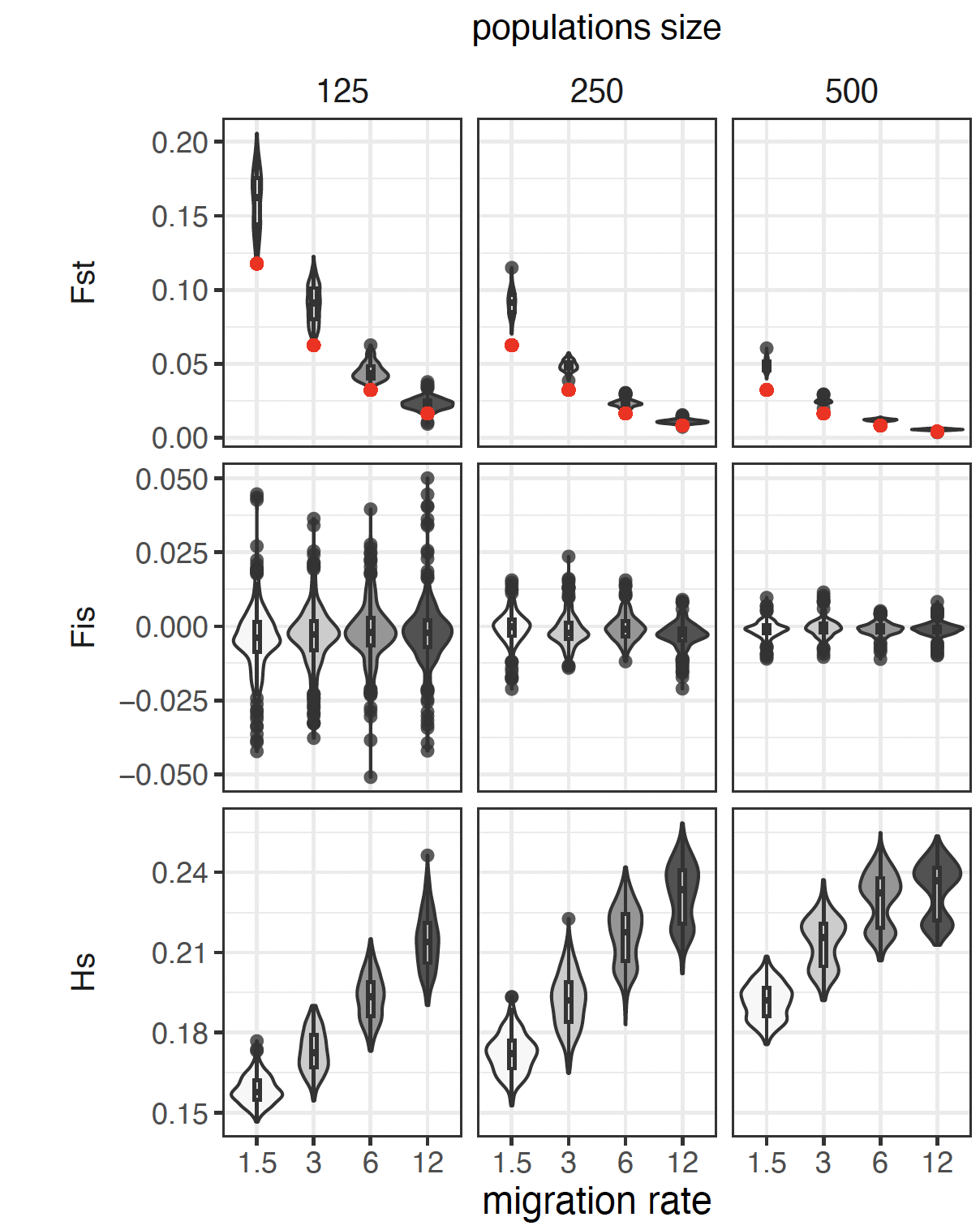
*

#### **Supplementary figure S1: Population genetic statistics across simulated datasets according to demographic parameters.**

Violin plots show the distribution of *F*_ST_ (first row), *F*_IS_ (second row) and gene diversity (HS, third row) values as a function of the simulated migration rate (x-axis) and population size (panels from left to right). Red points indicate the expected *F*_ST_ values under a Wright-Fisher island model (*F*_ST_ = 1 / (4Nm + 1)), shown here for reference only.


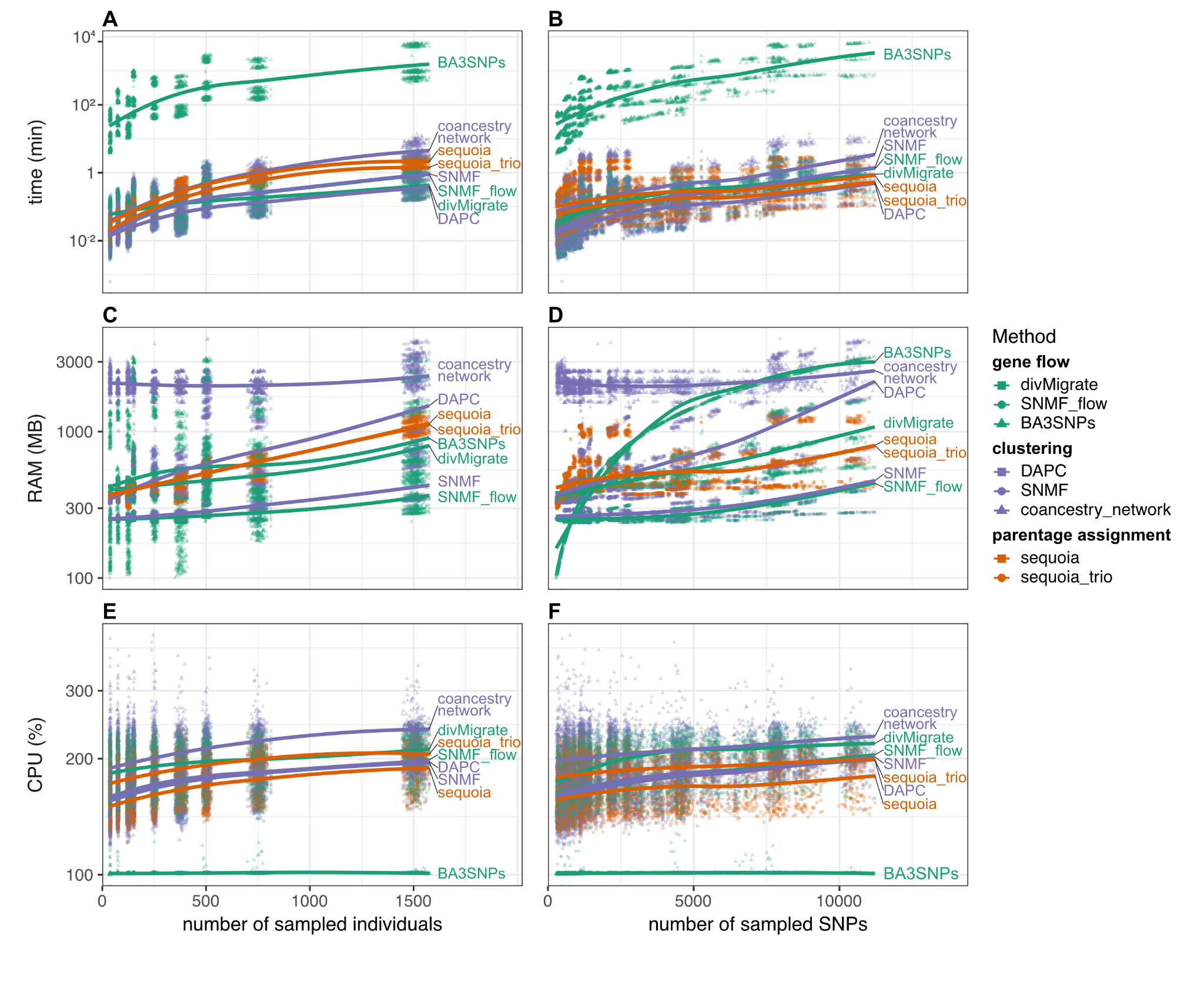


**Supplementary figure S2: Detailed resource usage of the eight tested methods as a function of data size.**

Resources usage of each method was recorded during each run (points). Runtime (A-B), memory (C-D) and CPU usage (E-F) are displayed as rows, from top to bottom, with y-axes in log scale. For CPU, 200% means that 100% of two cores are used. Results are shown as a function of (x-axes) the number of sampled individuals (A, C, E) or the number of SNPs (B, D, F). A polynomial regression line helps track the trend for each method. Colors indicate the broad method category: green for gene flow, purple for clustering, and orange for parentage assignment. Within each category, the point shapes differentiate methods.


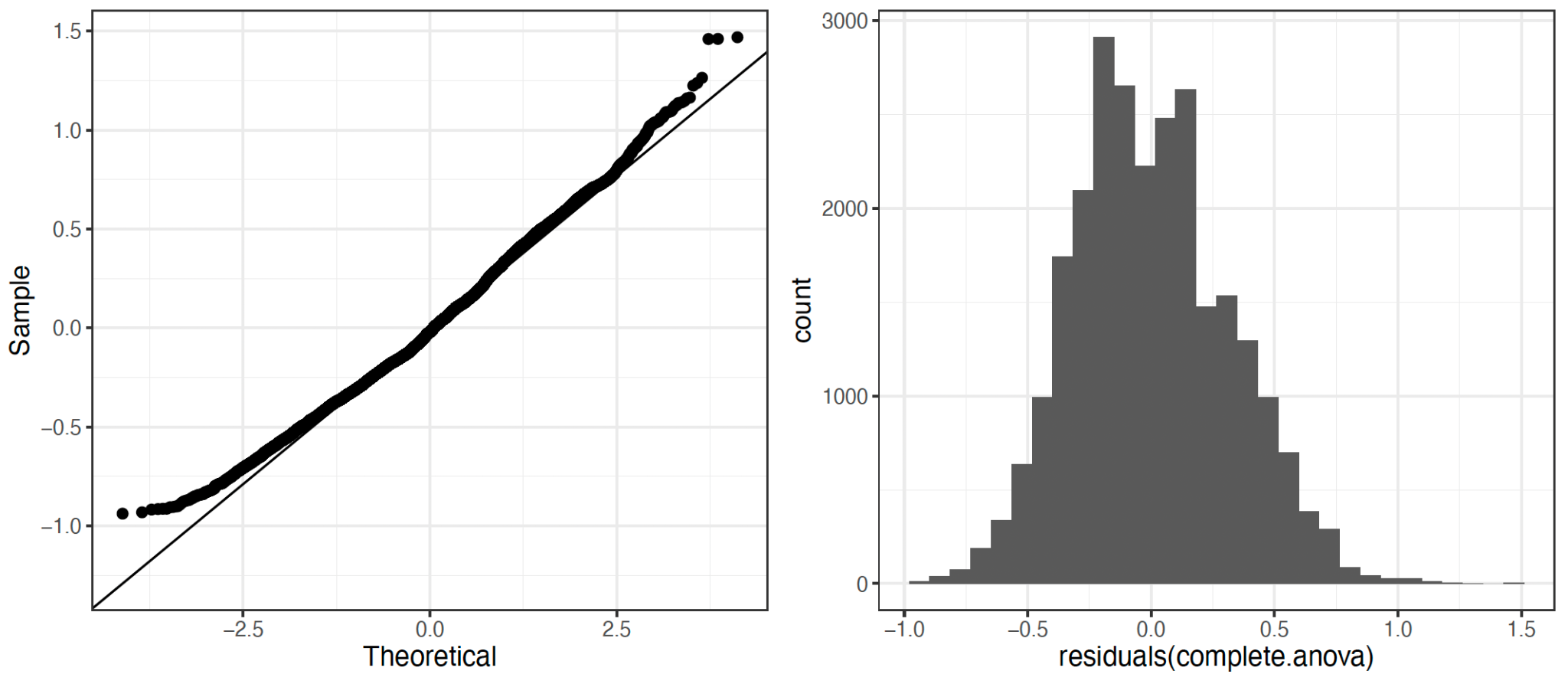


**Supplementary figure S3: Residual diagnostics of the ANOVA model.**

Quantile–quantile (QQ) plot (left) and histogram (right) of model residuals showing no major deviation from normality.


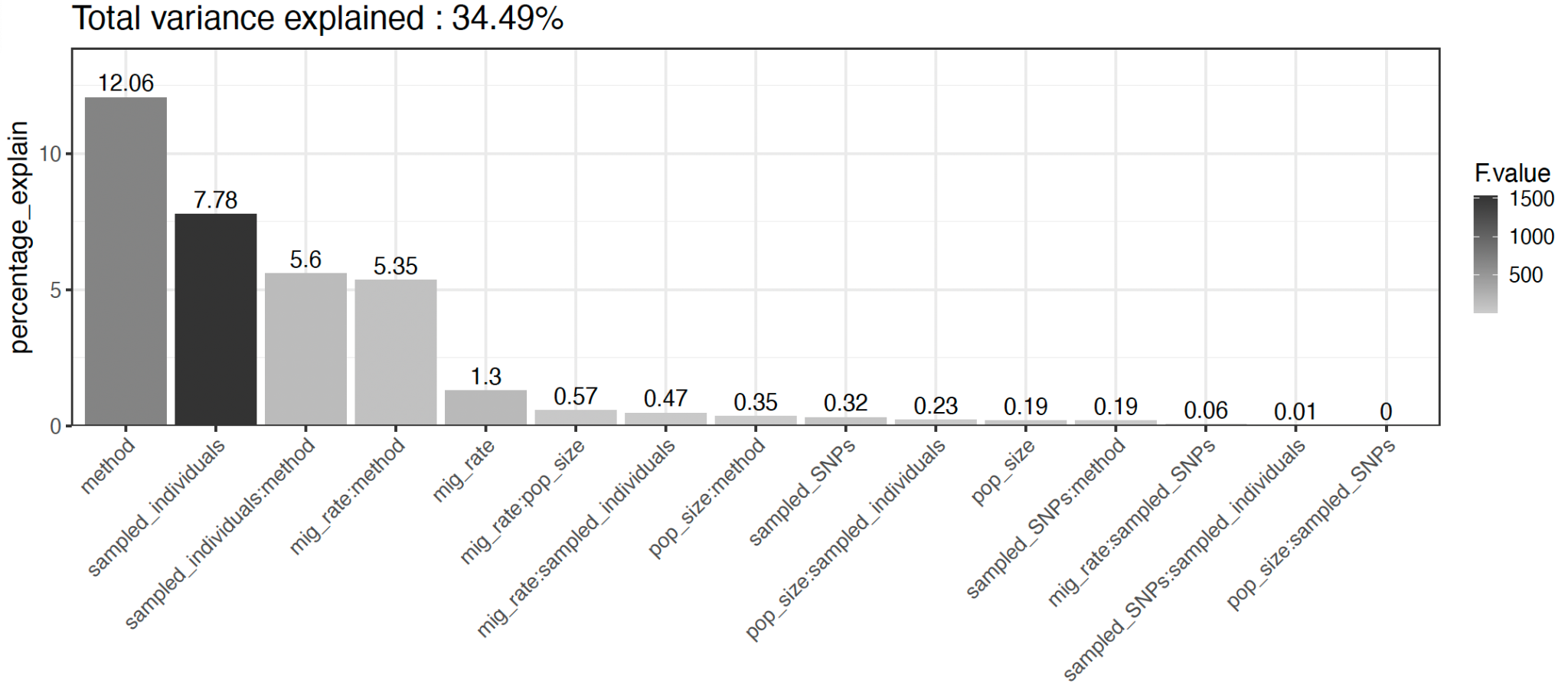


**Supplementary figure S4: Relative contribution of model terms to the variance explained by the ANOVA on mismatch (δ).**

Bars represent the percentage of variance explained by each main effect and interaction term, computed from the ANOVA sums of squares.

**
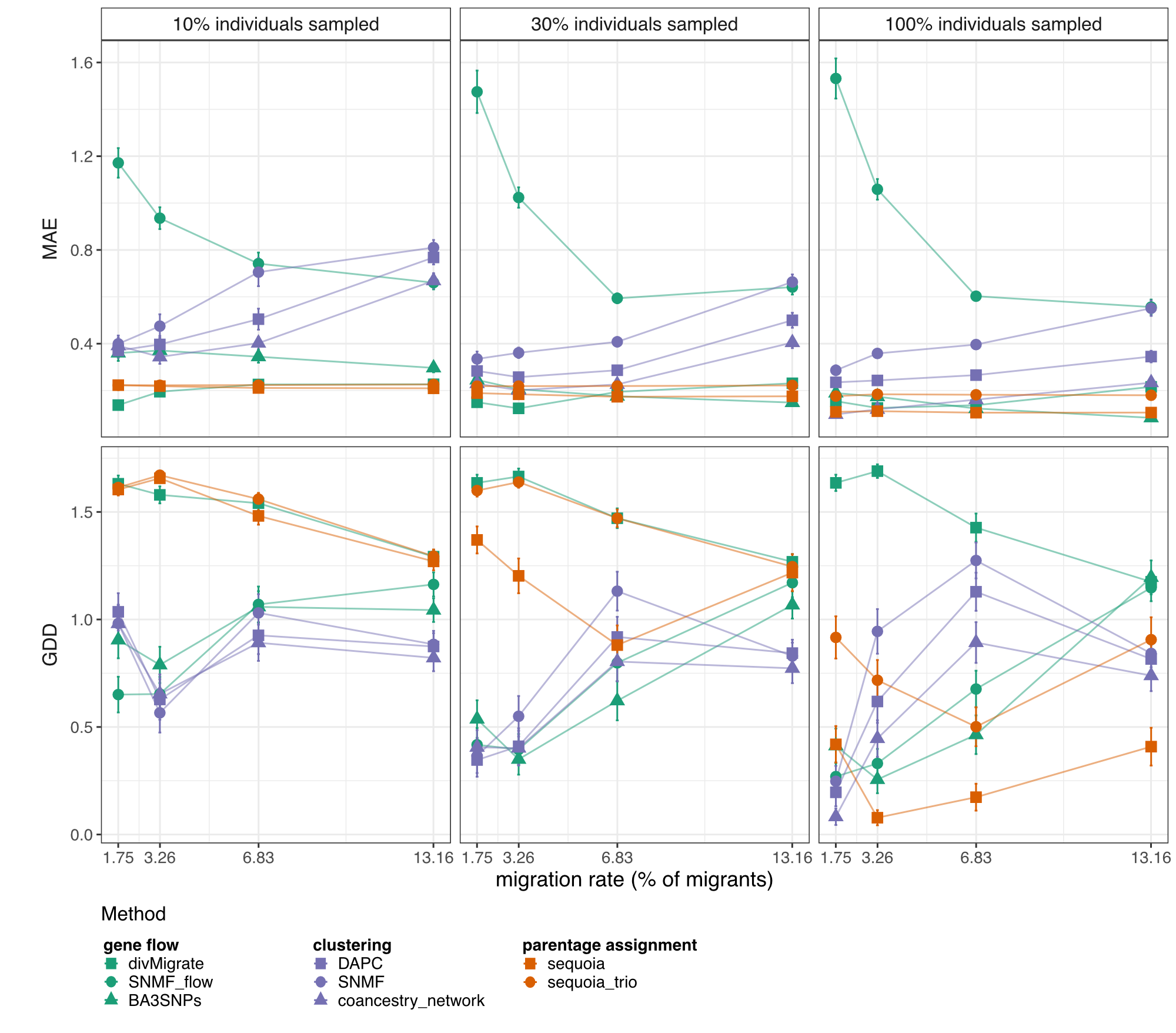
**

#### **Supplementary figure S5: Two components of the mismatch (δ) across sampling rates of individuals and migration rates.**

A decomposition of δ presented in Fig. 5 into its two components is shown: Mean Absolute Error (MAE) on the first row and Graph Diffusion Distance on binary network (GDD) on the second. As in Fig. 5, the values of the two metrics (y-axis) are displayed as a function of migration rate (x-axis) and percentage of sampled individuals (panels from left to right). Mean metric value of each method across the 270 simulations is represented by a point, and its 95% confidence interval by a bar. Colors represent broad method categories (green for gene flow, purple for clustering, and orange for parentage assignment) and point shapes represent individual methods.

#### **Supplementary table 1: Demographic and genetic parameters used for data simulation.**

The first column provides the name of each parameter, while the second column shows the corresponding value, range of values, or conditions applied.

| **Demographic parameters** | |
| --- | --- |
| Maintained sex ratio | 0.5 |
| Dispersive stage | between 0 et 1 year |
| Mortality before 1 year | 0.98 |
| Mortality between 1 and 19 years | 0.1 |
| Mortality at 20 years | 1 |
| Migration rate | 1.5%, 3%, 6%, or 12% |
| Reproduction conditions | Between individuals of ages above 2 years old of different sexes in the same population |
| Reproduction rate | Proportional to age with a factor 10 |
| **Genetic parameters** | |
| Number of simulated chromosomes | 14 |
| Mutation rate / nucleotide / reproductive event | 10^-7^ |
| Recombination rate | 10^-8^ |

**Supplementary table 2: Parameters used for connectivity analyses.**

The first column lists each method included in the study. The second column describes the parameters of each method, while the third column indicates the corresponding value, setting, or condition applied during the analyses.

| **Method** | **Parameter** | **Value / Description** |
| --- | --- | --- |
| BA3-SNPs | Mixing parameters | Automatically adjusted via BA3-SNPs-autotune |
|  | seed | 10 |
|  | iterations (MCMC generations) | 1000000 |
|  | sampling (Sampling interval for mcmc) | 100 |
|  | burnin (Burnin length for MCMC) | 100000 |
| divMigrate | boots | 10 |
|  | Function used | Modified version of divMigrate (divRsity R package), avoided normalization estimated number of migrants instead |
|  | plot_network | False |
| sNMF (LEA) | Function used | snmf (LEA R package) |
|  | Number of ancestral pops (K) | 3 |
|  | Number of runs (repetitions) | 10 |
|  | entropy | True |
|  | alpha | 10 |
|  | ploidy | 2 |
|  | percentage | 0.05 |
|  | seed | -1 |
|  | Run selection | Lowest cross-entropy |
| DAPC | Function used | find.clusters (adegenet R package) |
|  | Number of clusters | 3 |
|  | Number of PCs retained | 100 |
|  | method | kmeans |
|  | n.iter | 1,00E+05 |
|  | n.start | 1,00E+01 |
|  | center | True |
|  | scale | False |
| COANCESTRY + Louvain | Relatedness estimator | Lynch & Ritland (1999) |
|  | Function used | coancestry (related R package) |
|  | lynchrd | 1 |
|  | wang, lynchli, ritland, quellergt, dyadml | 0 |
|  | allow.inbreeding | False |
|  | Edge filtering for 3 clusters | Remove low-weight edges progressively |
|  | Community detection algorithm | Louvain |
|  | Function used | cluster_louvain (igraph 2.0.3 R package) |
|  | resolution | 1 |
|  | weights | NULL (weights of the input graph are considered insted) |
| Sequoia | Selected SNPs (max 1000) | 1000 top SNPs with highest minor allel frequency |
|  | Function used | GetMaybeRel (sequoia R package) |
|  | Tfilter | -4 |
|  | Mode | Parent-offspring |
|  | SeqList, Pedigree, LifeHistData, AgePrior | NULL |
|  | Module | par (Parent-offspring) |
|  | Complex | full |
|  | Herm | no |
|  | Err | 1,00E-04 |
|  | ErrFlavour | version2.9 |
|  | Tassign | 0.5 |
|  | Tfilter | -4 |
|  | MaxPairs | 7 * nrow(GenoM) |
